# Supplementary material for: A whole-animal phenotypic drug screen identifies suppressors of atherogenic lipoproteins
Source: eLife. 2026 Aug 27;14:RP105314. doi: 10.7554/eLife.105314 (PMC13521566; doi:10.7554/eLife.105314)
Supplement: Supplementary file 2 [file elife-105314-code1.zip › Output_data/rdu14_LP_level.pdf]

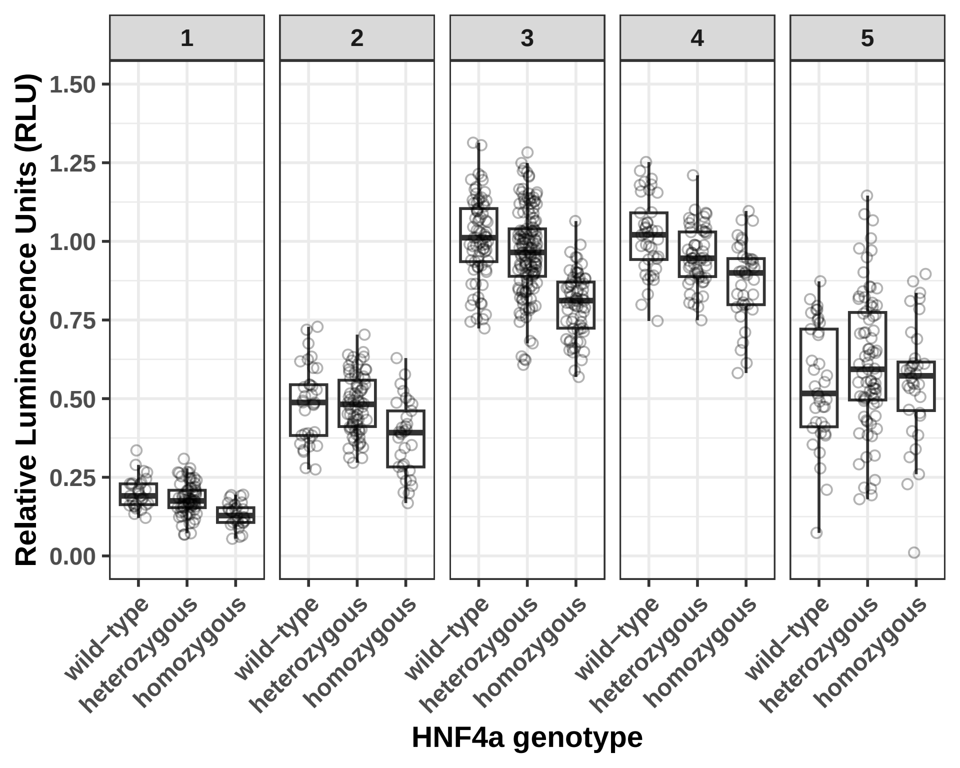

1
2
3
4
5
Relative Luminescence Units (RLU)
1.50
1.25
1.00
0.75
0.50
0.25
0.00
wild−type
heterozygous
homozygous
HNF4a genotype
